# An epigenetic score for BMI based on DNA methylation correlates with poor physical health and major disease in the Lothian Birth Cohort

**DOI:** 10.1038/s41366-018-0262-3

**Published:** 2019-03-06

**Authors:** Olivia K. L. Hamilton, Qian Zhang, Allan F. McRae, Rosie M. Walker, Stewart W. Morris, Paul Redmond, Archie Campbell, Alison D. Murray, David J. Porteous, Kathryn L. Evans, Andrew M. McIntosh, Ian J. Deary, Riccardo E. Marioni

**Affiliations:** 1 Centre for Cognitive Ageing and Cognitive Epidemiology, University of Edinburgh, Edinburgh, EH8 9JZ UK; 2 Centre for Clinical Brain Sciences, University of Edinburgh, Edinburgh, EH16 4SB UK; 3 Institute for Molecular Bioscience, University of Queensland, Brisbane, QLD Australia; 4 Queensland Brain Institute, University of Queensland, Brisbane, QLD Australia; 5 Centre for Genomic and Experimental Medicine, Institute of Genetics and Molecular Medicine, University of Edinburgh, Edinburgh, EH4 2XU UK; 6 Aberdeen Biomedical Imaging Centre, Lilian Sutton Building, University of Aberdeen, Foresterhill, Aberdeen, AB25 2ZD UK; 7 Division of Psychiatry, University of Edinburgh, Royal Edinburgh Hospital, Edinburgh, EH10 5HF UK; 8 Department of Psychology, University of Edinburgh, Edinburgh, Scotland UK

**Keywords:** Genetics, Cardiovascular diseases, Risk factors

## Abstract

**Background:**

The relationship between obesity and adverse health is well established, but little is known about the contribution of DNA methylation to obesity-related health outcomes. This study tests associations between an epigenetic score for body mass index (BMI) and health-related, cognitive, psychosocial and lifestyle outcomes in the Lothian Birth Cohort 1936. This study also tests whether these associations are independent of phenotypic BMI.

**Method:**

Analyses were conducted using data from the Lothian Birth Cohort 1936 (*n* = 892). Weights for the epigenetic BMI score were derived using penalised regression on methylation data from unrelated Generation Scotland participants (*n* = 2562). Associations were tested for replication in an independent sample: the Lothian Birth Cohort 1921 (*n* = 433).

**Results:**

A higher epigenetic BMI score was associated with higher BMI (*R*^2^ = 0.1), greater body weight (*R*^2^ = 0.06), greater time taken to walk 6 m, poorer lung function and poorer general physical health (all *R*^2^ = 0.02), greater levels of triglycerides (*R*^2^ = 0.09), greater %total HbA1c (*R*^2^ = 0.06), lower levels of high-density lipoprotein cholesterol (HDL; *R*^2^ = 0.08), higher HDL ratio (HDL/total cholesterol; *R*^2^ = 0.03), lower health-related quality of life, physical inactivity, and greater social deprivation (all *R*^2^ = 0.02). The epigenetic BMI score (per SD) was also associated with type 2 diabetes (OR 2.17, 95% CI 1.67, 2.84), cardiovascular disease (OR 1.45, 95% CI 1.24, 1.71) and high blood pressure (OR 1.30, 95% CI 1.13, 1.49; all *p* < 0.00026 after Bonferroni correction). Associations were replicated for BMI (*R*^2^ = 0.06), body weight (*R*^2^ = 0.04), health-related quality of life (*R*^2^ = 0.02), HbA1c (*R*^2^ = 0.07) and triglycerides (*R*^2^ = 0.07; all *p* < 0.0045 after Bonferroni correction).

**Conclusions:**

We observed and replicated associations between an epigenetic score for BMI and variables related to poor physical health and metabolic syndrome. Regression models with both epigenetic and phenotypic BMI scores as predictors accounted for a greater proportion of variance in all outcome variables than either predictor alone, demonstrating independent and additive effects of epigenetic and phenotypic BMI scores.

## Introduction

Body mass is a complex trait that demonstrates a high degree of variance in the general population [1]. It is determined by the contribution of genetic, lifestyle, physiological and psychosocial factors, but together these factors fail to fully explain the inter-individual variation in the most widely used clinical measure of body mass, the body mass index (BMI; kg/m^2^). Through recent advances in genomic analysis, we are now able to test whether obesity-related adverse health and disease may, in part, influence or be influenced by epigenetic changes, with the ultimate aim of more accurately predicting health outcomes.

Several recent studies have suggested links between BMI and DNA methylation: epigenome-wide association studies (EWAS) have identified CpG sites associated with BMI at loci involved in lipid and lipoprotein metabolism, blood lipid levels and inflammatory pathways [2–4]. Shah and colleagues [5] also found that methylation profiles associated with BMI accounted for 6.9% of the variance in BMI, independently of genetic profiles (polygenic scores), in a group of 1366 individuals from the Lothian Birth Cohort (LBC), which is the sample being studied in the present report.

A growing body of research examining the associations between DNA methylation and health assumes that exposures to adverse environmental stimuli induce epigenetic changes, which increase a genetically mediated risk of disease [6]. If it is the case that differential methylation profiles contribute to the risk of adverse health, there is potential utility in using epigenetic data in the prediction of health outcomes. In the current study, we test whether methylation profiles associate with adverse health outcomes. We examine (1) cross-sectional associations between an epigenetic score for BMI and health-related, cognitive, psychosocial and lifestyle outcomes, and (2) whether an epigenetic BMI score accounts for variance in these outcome variables independently of phenotypic BMI.

## Research design and methods

### The Lothian Birth Cohorts 1921 and 1936

The Lothian Birth Cohorts 1921 (LBC1921) and 1936 (LBC1936) are follow-up studies of the Scottish Mental Surveys of 1932 and 1947, respectively [7, 8]. These surveys tested the intelligence of 87,498 children (in 1932) and 70,805 children (in 1947) in Scotland at the age of 11 years, using the Moray House Test number 12. Approximately 60 years later, individuals who had taken the original tests and were living in Edinburgh and the Lothians were contacted, of whom 550 were recruited to the LBC1921 and 1091 to the LBC1936. Members of the LBC have since provided a wealth of cognitive, neuropsychological, psychosocial, biological (including genomic and other 'omics) and neuroimaging data longitudinally as part of the LBC study, which is ongoing [7, 8]. Recruitment and testing of the LBC1921 and LBC1936 have been described elsewhere [9, 10].

### Ethics

Ethical permissions for the LBC1921 were obtained from the Lothian Research Ethics Committee (LREC/1998/4/183). Permissions for the LBC1936 were obtained from the Multi-Centre Research Ethics Committee for Scotland (MREC/01/0/56) and the Lothian Research Ethics Committee (LREC/2003/2/29). Written informed consent was obtained from all participants.

### DNA methylation

DNA methylation data were assessed in whole blood samples from the LBC1921 and LBC1936 using the Illumina HumanMethylation450 BeadChip (Illumina Inc., San Diego, CA, USA). Full details of sample preparation and methylation typing have been reported previously [11]. In brief, background correction was performed and quality control was used to remove probes with a low detection rate (*p* > 0.01 for >5% of samples), low quality (manual inspection), low call rate (*p* < 0.01 for <95% of probes), and samples with a poor match between genotypes and single nucleotide polymorphism (SNP) control probes, and with incorrectly predicted sex.

The regression weights for the LBC1921 and LBC1936 BMI epigenetic signatures were derived from an independent cohort—Generation Scotland: the Scottish Family Health Study (GS; [12, 13]). Full details are provided in Supplementary Appendix 1.

### Phenotypic data

Continuous outcome measures formed six categories:

1. Physical health
  Physical health variables included height (cm), weight (kg), BMI (kg/m^2^), time taken to walk 6 m (s), grip strength (kg; measured using a North Coast Hydraulic Hand Dynamometer, JAMAR: Lafayette, Indiana, USA) and lung function (litres; forced expiratory volume in 1 s measured using a Micro Medical Spirometer). A measure of general physical health was also calculated using the first unrotated component of a principal component analysis of 6m walk time, grip strength and lung function.
2. Mental health
  Recent mood state was measured using the Hospital Anxiety and Depression Scale (HADS; [14]). The scale includes seven items for anxiety and seven items for depression and has a maximum score of 21, with scores of 11 or over indicating probable anxiety or depression.
3. Cognitive ability
  Variables measuring cognitive ability included the following subtests of the Wechsler Adult Intelligence Scale III (WAIS; [15]): Symbol Search and Digit Symbol Coding to assess speed of information processing, Matrix Reasoning to assess non-verbal reasoning, Letter-Number Sequencing and Backward Digit Span to assess working memory, and Block Design to assess constructional ability. The National Adult Reading Test (NART; [16]) was included as a measure of prior (or crystallised) cognitive ability. Finally, a measure of general fluid intelligence was calculated using the first unrotated component of a principal components analysis of the WAIS-III subtests described above.
4. Psychosocial factors
  Personality traits were measured using the 50-item version of the International Personality Item Pool questionnaire (IPIP; [17]). This questionnaire consists of ten items for measuring five personality factors: Extraversion, Agreeableness, Conscientiousness, Emotional Stability, and Intellect. We also included the World Health Organisation Quality of Life assessment (WHOQOL-BREF; [18]). This questionnaire produces scores for four domains related to quality of life: physical health, psychological, social relationships, and environment.
5. Bloods and biomarkers
  Blood samples were taken to assess glycated haemoglobin (%total HbA1c), fibrinogen (g/l), C-reactive protein (mg/l), triglycerides (mmol/l), total cholesterol (mmol/l) and high-density lipoprotein cholesterol (HDL; mmol/l). A HDL ratio was calculated by dividing HDL by total cholesterol. Total cholesterol levels were adjusted for individuals on lipid-lowering medications. Systolic and diastolic blood pressure, both sitting and standing, was measured at our research facility with an Omron 705IT monitor (Milton Keynes, UK). Blood pressure scores were adjusted for individuals taking antihypertensive medications.
6. Lifestyle factors

Social deprivation was measured using the Scottish Index of Multiple Deprivation (SIMD; [19]). The SIMD ranks geographical areas in Scotland based on current income, employment, health, education, skills and training, geographic access to services, housing and crime. The SIMD provides a standardised measure of relative deprivation throughout Scotland. In the present study, a lower score indicates a greater level of deprivation. Units of alcohol consumed per week and physical activity were reported by participants during a structured interview with a member of the LBC research team. Physical activity was coded as follows: (1) “household chores” (2) “walking etc. 1–2 times a week”, (3) “walking etc. several times a week”, (4) “exercise 1–2 times a week”, (5) “exercise several times a week”, (6) “keep-fit/heavy exercise/sport several times a week”. Dietary data were derived from the Scottish Collaborative Group 168-item Food Frequency Questionnaire, version 7.0 [20, 21], in which participants are asked to indicate how much of certain foods they have consumed in the past 2–3 months. The dietary patterns measured in the current study are Mediterranean diet, health-aware diet, traditional diet, and sweet foods. Dietary patterns were extracted via principal components analysis with orthogonal rotation from all FFQ items. Further details on the construction of the dietary patterns and food types associated with each pattern are available in Corley et al. [22].

Participants reported their disease history during a structured interview with a member of the LBC research team. Self-reported disease history variables were binary and included: high blood pressure, type 1 diabetes mellitus, type 2 diabetes mellitus, high cholesterol, cardiovascular disease, leg pain, poor blood circulation, stroke, neoplasm, health issues related to the thyroid, Parkinson’s disease, arthritis and allergies.

Descriptive statistics for all continuous outcome variables from LBC1936 and LBC1921 are presented in Table S1. Further information on data collection and laboratory procedures can be found in published protocols for the first waves of data collection for the LBC1921 [9] and LBC1936 [10].

### Statistical analyses

Linear regression models were used to investigate the relationship between the epigenetic BMI score and outcome variables. For each model, the *R*^2^ statistic represents the proportion of variance in the outcome variable that can be accounted for by the epigenetic BMI score (see model 1), phenotypic BMI score (model 2), and both epigenetic BMI and phenotypic BMI scores together (model 3). To estimate the proportion of variance accounted for by these predictors without the effect of covariates, the *R*^2^ statistic represents the difference between the *R*^2^ of the null model (see below) and the *R*^2^ of models 1, 2 and 3, respectively. Age at the time of testing and sex were included as covariates in all models. Height was included as an additional covariate in models for time taken to walk 6 m, forced expiratory volume (lung function) and general physical health. The models are as follows:

**Model 1:** variable of interest ~ epigenetic BMI score + age at time of testing + sex.

**Model 2**: variable of interest ~ phenotypic BMI + age at time of testing + sex.

**Model 3:** variable of interest ~ epigenetic BMI score + phenotypic BMI + age at time of testing + sex.

**Null model:** variable of interest ~ age at time of testing + sex.

Logistic regressions were conducted for self-reported disease history variables as these had binary outcomes (disease/no disease). Similar to the models above, logistic regressions were carried out for each variable to test whether epigenetic BMI, phenotypic BMI, and an additive model with both epigenetic BMI score and phenotypic BMI as predictor variables, were associated with disease history. For all analyses carried out using LBC1936 data, a Bonferroni corrected level of significance was used (*p* < 0.05/*n*_tests = 0.00026). Analyses were carried out in R version 3.4.0 [23].

### Sensitivity analysis

For variables that associated with epigenetic BMI, we carried out sensitivity analyses, by including a polygenic score for BMI (for details, see Supplementary Appendix 2) as a predictor in the additive model together with epigenetic BMI score and phenotypic BMI (model 4).

**Model 4:** variable of interest ~ epigenetic BMI score + phenotypic BMI + polygenic BMI score + age at time of testing + sex.

### Replication analyses in the LBC1921

Significant associations between epigenetic BMI score and outcome variables were tested for replication in an independent sample, the LBC1921. Prior to testing, we carried out power analyses based on the effect sizes of associations in the LBC1936, to assess whether the sample size for each variable in the LBC1921 was large enough to detect a significant effect (80% power, *α* = 0.05). For all analyses carried out using LBC1921 data, a Bonferroni corrected level of significance was used (*p* < 0.05/*n*_tests = 0.0045).

## Results

Analyses of the LBC1936 included 892 individuals (female, *n* = 440, 49.3%), with a mean age of 69.5 years.

### Epigenetic BMI score is associated with outcomes related to poor physical health, biomarkers of metabolic syndrome and social deprivation

Only associations with a *p* value < 0.00026 (i.e. after Bonferroni correction) are presented here—full results are presented in Table S2.

The results of the linear regression models showed that epigenetic BMI was strongly associated with phenotypic BMI (*p* < 2 × 10^–16^) and accounted for 10% of its variation. Increases in epigenetic BMI score also correlated with poorer performance on other physical health variables: as epigenetic BMI score increased by 1 SD, time taken to walk 6 m increased by 0.14 s (*p* = 2.5 × 10^−05^, *R*^2^ = 0.02), body weight increased by 0.24 kg (*p* = 4.1 × 10^−16^, *R*^2^ = 0.06), forced expiratory volume decreased by 0.13 l (*p* = 1.1 × 10^–07^, *R*^2^ = 0.02), and general physical health decreased by 0.13 SDs (*p* = 1 × 10^−07^, *R*^2^ = 0.02).

Regarding blood and biomarker variables, epigenetic BMI score was associated with higher levels of %total HbA1c (*p* = 1.1 × 10^−13^, *R*^2^ = 0.06), triglycerides (*p* < 2 × 10^−16^, *R*^2^ = 0.09), and HDL ratio (HDL cholesterol/total cholesterol; *p* **=** 2.4 × 10^−07^, *R*^2^ = 0.03), and lower levels of HDL cholesterol (HDL; *p* < 2 × 10^−16^, *R*^2^ = 0.08).

Finally, epigenetic BMI score was associated with lower physical health-related quality of life (*p* = 4 × 10^−5^, *R*^2^ = 0.02), lower scores on the Scottish Index of Multiple Deprivation (*p* = 9.9 × 10^−05^, *R*^2^ = 0.02), and lower levels of self-reported physical activity (*p* = 1.6 × 10^−04^, *R*^2^ = 0.02).

### Epigenetic BMI score and phenotypic BMI account for independent proportions of variance

Phenotypic BMI was associated with greater time taken to walk 6 m (*p* = 2.6 × 10^−16^, *R*^2^ = 0.07), greater body weight (*p* < 2 × 10^−16^, *R*^2^ = 0.62), poorer general physical health (*p* = 7.1 × 10^−11^, *R*^2^ = 0.02), higher levels of %total HbA1c (*p* = 3.6 × 10^−13^, *R*^2^ = 0.06), higher levels of triglycerides (*p* = 2.2 × 10^−15^, *R*^2^ = 0.07), higher HDL ratio (*p* = 3.3 × 10^−07^, *R*^2^ = 0.03), lower levels of HDL cholesterol (*p* **<** 2 × 10^−16^, *R*^2^ = 0.08), poorer physical health-related quality of life (*p* = 1.3 × 10^−14^, *R*^2^ = 0.07), and lower levels of self-reported physical activity (*p* = 4.4 × 10^−07^, *R*^2^ = 0.03). With the exception of triglycerides, phenotypic BMI accounted for a greater or equal amount of variance of all outcome variables, relative to epigenetic BMI.

**Fig. 1:**
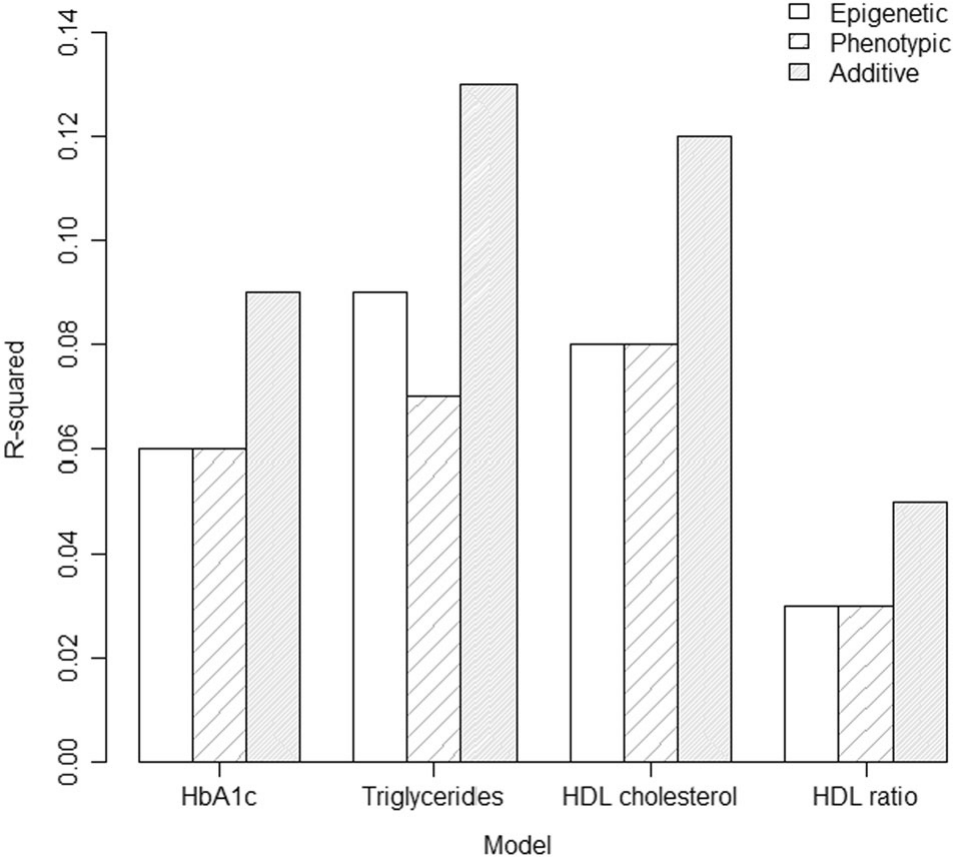
Plot of *R*^2^ statistics from linear regression analyses in which epigenetic BMI, phenotypic BMI and epigenetic + phenotypic BMI, respectively, associated with biomarker variables at *p* < 0.00026. The plots demonstrate the general pattern observed, with the additive model accounting for a greater proportion of variance in the outcome variable  than models including epigenetic BMI score only, or phenotypic BMI only

Linear regression models with both epigenetic and phenotypic BMI scores as predictor variables, demonstrated that epigenetic and phenotypic BMI explain partially independent proportions of variance in the outcome variable. This pattern was most striking for biomarker variables related to metabolic syndrome— models that demonstrated associations with these biomarkers are presented in Fig. 1.

### Epigenetic BMI score is associated with self-reported disease history

The epigenetic BMI score (per SD) was positively associated with type 2 diabetes (OR 2.17, 95% CI 1.67, 2.84), cardiovascular disease (OR 1.45, 95% CI 1.24, 1.71), and high blood pressure (OR 1.30, 95% CI 1.13, 1.49). Phenotypic BMI was associated with type 2 diabetes (OR 1.83, 95% CI 1.45, 2.31) and high blood pressure (OR 1.55, 95% CI 1.34, 1.80). In an additive model with both epigenetic and phenotypic BMI scores as predictors, epigenetic BMI score was associated with type 2 diabetes (OR 1.92, 95% CI 1.45, 2.55) and cardiovascular disease (OR 1.39, 95% CI 1.18, 1.65) - all associations significant at *p* < 0.00026. Full results of these logistic regression models are presented in Table S3.

### Replication of associations with epigenetic BMI score in LBC1921

Analyses of the LBC1921 included 433 individuals (female, *n* = 263, 60.7%), with a mean age of 79.1 years. Where possible, variables that associated with epigenetic BMI score in the LBC1936 (*n* = 15) were tested for replication in the LBC1921. Some variables were not available in the LBC1921 (*n* = 5) and we did not have sufficient statistical power to detect the effects for several others (*n* = 2). Only associations with a *p* value < 0.0045 (i.e. after Bonferroni correction) are presented in Table 1—full results are presented in Tables S4 and S5. In the LBC1921, associations were found between epigenetic BMI score and body weight, BMI, physical health-related quality of life, %total HbA1c, and triglyceride levels.

**Table 1:** Replication of associations between epigenetic BMI and outcome variables in the LBC1921

		Epigenetic BMI			
	*n*	Standardised *β*	SE	Raw *p* =	*R* ^2^
*Physical health*					
Weight (kg)	433	0.198	0.04	**2.71** × **10**^−**06**^	0.04
BMI (kg/m^2^)	432	0.246	0.05	**3.31** × **10**^−**07**^	0.06
*Psychosocial*					
WHOQOL: physical health	378	−0.162	0.05	**0.002**	0.02
*Bloods and biomarkers*					
HbA1c (%total)	379	0.270	0.05	**9.72** × **10**^−**08**^	0.07
Triglyceride (mmol/L)	421	0.262	0.05	**3.22** × **10**^−**08**^	0.07

Raw *p* values lower than the Bonferroni-corrected level of significance (*α* = 0.0045) are presented in bold

### A polygenic score for BMI did not increase *R*^2^ of the additive models in LBC1936

Tables S6 and S7 present the results of the linear and logistic regression models in which a polygenic score for BMI was included as a predictor variable alongside epigenetic BMI score and phenotypic BMI. In these additive models, a polygenic BMI score did not associate with any of the variables tested. In a linear regression model with both epigenetic BMI score and polygenic BMI score as predictor variables, polygenic BMI demonstrated a strong association with phenotypic BMI (*p* < 2 × 10^−16^) and together, predictors accounted for 18% of the variation in phenotypic BMI.

## Discussion

Using DNA methylation data, we constructed an epigenetic score for BMI to test its association with health, cognitive and lifestyle outcomes in the LBC1936. We found that a higher  epigenetic score for BMI is associated with poorer physical health (body weight, time taken to walk 6 m, lung function and general physical health), higher levels of HbA1c and triglycerides, higher HDL ratio, lower HDL cholesterol, lower physical health-related quality of life, lower self-reported physical activity and greater social deprivation. We replicated several of these associations (BMI, weight, physical health-related quality of life, HbA1c and triglycerides) in an independent, older sample, the LBC1921. Our analysis of the LBC1936 also showed that epigenetic BMI score is associated with self-reported type 2 diabetes, cardiovascular disease and high blood pressure: health outcomes for which obesity is a major risk factor. An association between an epigenetic score for BMI and type 2 diabetes, identified via clinical diagnosis or HbA1c ≥6.5%, has previously been reposted by Wahl et al. [2].

In almost all analyses, phenotypic BMI accounted for a greater proportion of variance in outcome variables than epigenetic BMI, but phenotypic and epigenetic BMI scores together accounted for a greater proportion of variance still. This additive effect suggests that epigenetic and phenotypic BMI scores account for at least partially independent proportions of variance. It is possible that some of these partially independent associations are driven by correlations between the CpG probes used to create the epigenetic BMI score, and the outcome variables of interest. This could mean that our epigenetic score for BMI is capturing the downstream effects of obesity, such as biological processes associated with poor metabolic health, which phenotypic BMI (a more ‘blunt’ measure of metabolic health) does not account for directly.

We found that an epigenetic score for BMI accounted for 10% of the variance in phenotypic BMI, a higher proportion than that reported by Shah et al. [5], whose epigenetic BMI score accounted for 6.9% of the variance in phenotypic BMI in the LBC1936. This improvement could be due to our larger discovery sample (2562 vs. 1366), and/or because weights for our epigenetic BMI score were calculated using a single regression model, with BMI residuals as the outcome and CpGs as predictors, rather than separate models for each CpG predictor (see Supplementary Appendix 1). Shah et al. also found that an epigenetic BMI score increased the phenotype prediction when combined with a genetic score for BMI from 8% to 14% [5]. By including both an epigenetic and a polygenic score for BMI as predictors in our linear regression model, we improved the proportion of variance in phenotypic BMI explained from 10% to 18%. However, our polygenic score for BMI did not associate with any outcome variables when included alongside epigenetic and phenotypic BMI scores as predictors in regression models. In the majority of these models, polygenic BMI score had little effect; associations between epigenetic BMI score and outcome variables remained, particularly for physical health and biomarker variables. It could be the case that the epigenetic BMI score encompasses the interaction between the polygenic BMI score and outcome variables implicated in biological processes related to obesity (as opposed to cognitive or lifestyle variables). If this is the case, there appears to be little benefit in the inclusion of a polygenic score for BMI alongside epigenetic BMI and phenotypic BMI scores, when testing associations with biological and physical health-related variables.

Our results demonstrate that BMI (and epigenetic BMI) is associated with obesity-related adverse health and thus, is a clinically relevant metric for measuring body mass. However, the BMI has been criticised due to its assumption that a low body mass equates to metabolic health. Dvorak et al. [24] found that BMI failed to distinguish between healthy individuals and individuals who were metabolically unhealthy, but of normal weight despite differences in total fat mass, body fat percentage, subcutaneous fat and visceral abdominal adiposity. It has also been shown that waist to height ratio (WHtR) and waist/height^0.5^ (WHT.5R) are better predictors of whole body fat percentage and visceral adipose tissue mass than BMI [25]. Despite this, the BMI continues to be widely used in clinical settings. A development upon this study would be to test whether an epigenetic score based on WHtR or WHT.5R can further increase the proportion of variance in obesity-related outcomes explained by epigenetic BMI.

That our replication sample (the LBC1921) was smaller than our discovery sample (the LBC1936) is a limitation of this study. While we were able to replicate five out of eight associations with epigenetic BMI score in the LBC1936, the smaller sample size of the LBC1921 prevented us from testing a greater number of associations due to a lack of power. A further limitation of this study is the inability to make strong directional inferences; it is impossible to tell whether differential DNA methylation is a mediator of the causal disease pathway, or occurs in response to disease processes. Recent progress has been made in this area using a Mendelian Randomisation approach [26], which takes SNPs as instruments for exposure variables, such as DNA methylation, in order to gain insight into the direction of causality. Longitudinal studies are another important method for gaining insight into the role of DNA methylation in the pathophysiology of obesity-related disease. Several studies have analysed DNA methylation from umbilical cord blood as a baseline measure for longitudinal epigenetic data. This is then analysed in tandem with longitudinal data on health-related outcomes in order to capture epigenetic changes that may predate disease onset [27]. These cohorts must have deep phenotyping of lifestyle and environmental factors (including prenatal factors such as maternal diet and smoking), which may confound epigenetic changes. Another developing line of research is investigating epigenetic changes associated with weight-loss interventions such as diet programmes or gastric bypass surgery [28, 29].

A recent study by Richmond et al. [30] combined several of these methods to investigate causality between BMI and methylation at *HIF3A* (a gene linked to metabolism and obesity, and associated with adiposity in multiple EWAS [31, 32]), including bidirectional Mendelian randomisation analysis, longitudinal analysis of *HIF3A* methylation, and analysis of association between maternal pregnancy BMI and *HIF3A* methylation in offspring at birth (using cord blood). The results across each method indicated that a higher BMI causally influenced higher *HIF3A* methylation, suggesting that alterations in DNA methylation are a downstream effect of obesity. Further longitudinal studies and studies of naturalistic interventions are needed to elucidate the direction of causal association between obesity and DNA methylation, and to provide mechanistic insight into the pathophysiology of obesity-related disease.

We have demonstrated that an epigenetic score for BMI is associated with poorer physical health, poorer health-related quality of life, biomarkers for metabolic syndrome, physical inactivity, social deprivation and major diseases for which obesity is a risk factor. We replicated the associations between BMI, body weight, HbA1c, triglycerides and physical health-related quality of life in an independent, older cohort. An epigenetic score for BMI based on DNA methylation increased the amount of variance in health outcomes accounted for by phenotypic BMI score alone, demonstrating the value in using epigenetic information for constructing more accurate risk prediction for obesity-related biomarkers, health conditions and disease.

## Electronic supplementary material

Supplementary Tables

Appendix 1
