# Supplementary material for: An epigenetic score for BMI based on DNA methylation correlates with poor physical health and major disease in the Lothian Birth Cohort
Source: Int J Obes (Lond). 2019 Mar 6;43(9):1795–802. doi: 10.1038/s41366-018-0262-3 (PMC6760607; doi:10.1038/s41366-018-0262-3)
Supplement: Supplementary file 2 — Appendix 1 [file 41366_2018_262_MOESM2_ESM.docx]

**An epigenetic score for BMI based on DNA methylation correlates with poor physical health and major disease in the Lothian Birth Cohort.**

**Appendix 1**. Generation Scotland and building the BMI Epigenetic Signature

Generation Scotland: the Scottish Family Health Study is a large population-based, family-structured prospective cohort. Full study details have been reported previously (1, 2).

**DNA Methylation**

Illumina EPIC array methylation data were collected on 5 200 participants. The raw methylation beta-values were quality checked using the R packages shinyMethyl (3) and watermelon (4). First, shinyMethyl was used to create a “QC plot”, which plots the log median intensity of the methylated signal against the log median intensity of the unmethylated signal for each array. Outlier samples on the QC plot were identified by visual inspection and excluded from the dataset. Subsequently, the pfilter function in wateRmelon was used to exclude poor-performing samples and probes. Samples were excluded if ≥ 1% sites had a detection *p-*value of > 0.05. Probes were removed from the dataset if: (i) they had more than 5 samples with a beadcount of less than 3; or (ii) ≥ 0.5% samples had a detection *p*-value of > 0.05. Finally, shinyMethyl’s sex prediction plot was used to exclude samples whose predicted sex differed from their recorded sex. After QC, the dataset comprised beta-values for 860 926 methylation loci for 5 101 participants. A subset of 2 562 unrelated individuals, with a relationship coefficient below 0.05 and with no shared nuclear environment, were considered for the analysis.

*Exclusions and data preparation*

Further exclusions were carried out for individuals with BMI measurements of ≤17 or ≥50. Residuals from a linear regression model of BMI on age, sex, and 10 genetic principal components (to control for population stratification) were carried forward to the penalised regression analysis.

To ensure the epigenetic signature for BMI was applicable to LBC, the GS methylation dataset was restricted to autosomal CpG sites that were present on both the EPIC and 450k arrays. CpG sites with missing values were excluded from the penalised regression model. 11 individuals had missing BMI values, 6 were excluded for having BMI < 17, and 3 for a BMI > 50. This left 385 396 CpGs from 2 562 individuals available for analysis, with a mean age 50.0 years and a mean BMI of 27.3 (female n=1571, 61.3%).

**Statistical analysis**

A penalised regression model with the BMI residuals as the outcome and the 385 396 CpGs as predictors was run using the glmnet package in R (5). Default settings for the cross-validation glmnet model were considered: 10-fold cross validation and alpha = 1 (LASSO penalty). The penalised regression model was run 100 times and the best-fit lambda values were extracted. The model was then re-run using the best lambda from these 100 runs to give the optimal solution.

**Results**

The best predictor from the LASSO model included non-zero weights for 400 CpG sites (see table S8 in supplementary materials for CpG sites and weights).

**Appendix 2**. Building the polygenic risk score for Body Mass Index

LBC1936 DNA samples were genotyped at the Wellcome Trust Clinical Research Facility using the Illumina 610-Quadv1 array (San Diego). Data preparation and quality control steps included exclusions based on relatedness, sex discrepancies, low SNP call rate, evidence of non-European descent. SNPs with a minor allele frequency >1% and a Hardy-Weinberg p-value >= 0.001. Full details have been reported previously (6).

Polygenic risk scores for BMI were calculated using the PRSice software program with LD clumping parameters set to R^2^>0.25 over 250kb sliding windows (7). Summary statistics for the SNP associations were taken from the GWAS analysis of Locke et al. (8). The LBC1936 polygenic scores were generated using all possible SNPs (P<1) from the discovery GWAS.

**REFERENCES**

1 Smith BH, Campbell H, Blackwood D, Connell J, Connor M, Deary IJ et al. Generation Scotland: the Scottish Family Health Study; a new resource for researching genes and heritability. BMC Med. Genet.*,* 2006;7:74.

2 Smith BH, Campbell A, Linksted P, Fitzspatrick B, Jackson C, Kerr SM et al. Cohort Profile: Generation Scotland: Scottish Family Health Study (GS:SFHS). The study, its participants and their potential for genetic research on health and illness. Int J Epidemiol, 2013;42(3):689-700.

3 Fortin JP, Fertig E, Hansen K. shinyMethyl: interactive quality control of Illumina 450k DNA methylation arrays in R. F1000Res 2014; 3:175.

4 Pidsley R, Wong CCY, Volta M, Lunnon K, Mill J, Schalkwyk LC. A data-driven approach to preprocessing Illumina 450K methylation array data. BMC Genomics 2013; 14:293.

5 Friedman J, Hastie T, Tibshirani R. Regularization Paths for Generalized Linear Models via Coordinate Descent. Jo Stat Softw 2010; 33(1), 1-22.

6 Davies G, Tenesa A, Payton A, Yang J, Harris SE, Liewald D et al. Genome-wide association studies establish that human intelligence is highly heritable and polygenic. Mol Psychiatry 2012; 16(10): 996-1005.

7 Euesden J, Lewis CM, O’Reilly PF. PRSice: Polygenic risk score software. Bioinformatics 2015; 31(9):1466-8.

8 Locke AE, Kahali B, Berndt SI, Justice AE, Pers TH, Day FR et al. Genetic studies of body mass yield new insights for obesity biology. Nature 2015; 518(7538): 197-206.
